# SRSF7 serves as a potential therapeutic target in acute myeloid leukemia

**DOI:** 10.1016/j.gendis.2025.101739

**Published:** 2025-06-26

**Authors:** Dantong Shang, Hongkai Zhu, Yulin Pu, Long Liang, Haodong Xu, Yue Sheng, Huifang Zhang

**Affiliations:** a Department of Hematology, The Second Xiangya Hospital, School of Life Sciences, Central South University, Changsha, Hunan 410011, China; b Department of Orthopaedics, The Second Xiangya Hospital, Central South University, Changsha, Hunan 410011, China; c Center for Medical Research, The Second Xiangya Hospital, Central South University, Changsha, Hunan 410011, China

Aberrant accumulation of myeloid stem cell precursors within the bone marrow caused acute myeloid leukemia (AML), leading to a disruption of normal hematopoiesis. Despite significant therapeutic advancements improving AML patient outcomes, up to 70% of individuals aged 65 or older succumb within the first year of diagnosis.^1^ Identifying new targets for AML treatment remains important. SRSF7, an important member of the family of serine/arginine-rich splicing factors (SRSFs), was characterized as a key regulator of mRNA export.^2^ A wealth of research has highlighted multifaceted contributions of SRSF7 to oncogenic mechanisms, but its role in AML progression is yet to be investigated.

Bioinformatics analysis of BloodSpot indicated significantly elevated SRSF7 expression in AML with various fusion genes compared with hematopoietic stem cells from healthy donors (Fig. 1A). In addition, our findings reveal that an elevated expression of SRSF7 predicted reduced overall survival in AML patients (Fig. S1A). We subsequently verified SRSF7 expression in AML cells. We found that SRSF7 protein was elevated in four AML cells with diverse cytogenetic abnormalities and AML patients compared with peripheral blood mononuclear cells derived from healthy donors (Fig. S1B). These findings indicate that SRSF7 could serve as a marker for AML development.

**Figure 1:**
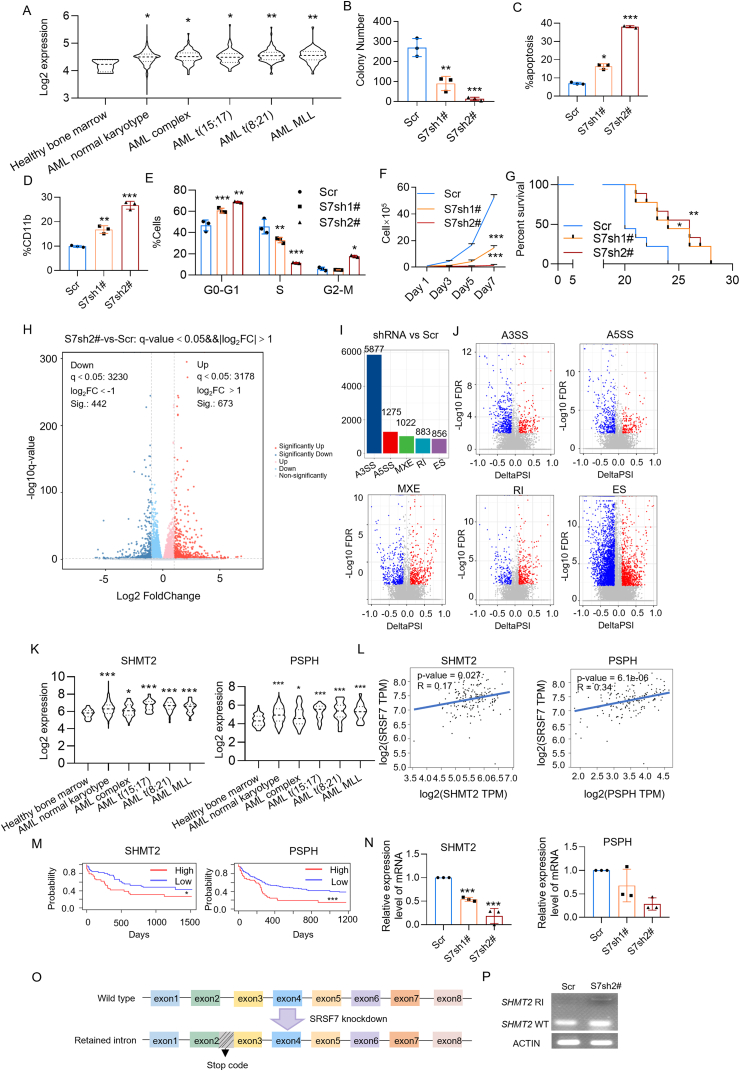
SRSF7 serves as a promising therapeutic target in acute myeloid leukemia (AML). **(A)***SRSF7* mRNA expression in the primary AML patients with different chromosome translocations compared with that of healthy donors (*n* = 4 in the healthy bone marrow group, *n* = 989 in the AML normal karyotype group, *n* = 87 in the AML complex group, *n* = 87 in the AML t(15; 17) group, *n* = 98 in the AML t(8; 21) group, and *n* = 58 in the AML MLL group). **(B)** Colony formation of MOLM-13 cells expressing control Scramble or SRSF7 shRNAs was analyzed. **(C)** Flow cytometry analyzed the apoptosis of MOLM-13 expressing control Scr or SRSF7 shRNAs. **(D)** Flow cytometry analyzed the ratio of CD11b-positive MOLM-13 cells expressing control Scr or SRSF7 shRNAs. **(E)** Flow cytometry analyzed the cell cycle progression of MOLM-13 cells expressing control Scr or SRSF7 shRNAs. **(F)** Growth curves of MOLM-13 cells expressing control Scr or SRSF7 shRNAs. One-way ANOVA. **(G)** Kaplan–Meier survival analysis was performed on immunodeficient mice transplanted with MOLM-13 cells (*n* = 9 in each group) expressing either control Scr or SRSF7 shRNAs. Log-rank test. **(H)** The volcano plot illustrating fold changes for differentially expressed genes in MOLM-13 cells with Scr or SRSF7 shRNA2# expression. Cells were collected three days post-viral infection, at which point all cell groups exhibited comparable viability. The experiments were conducted in triplicate. **(I)** The changes in mRNA splicing events in MOLM-13 cells following SRSF7 knockdown: A3SS, A5SS, MXE, RI, and ES. **(J)** The volcano plot illustrating the changes in A3SS, A5SS, MXE, RI, and ES splicing events following SRSF7 knockdown, with percent spliced-in (PSI) ≥ 0.1 and false discovery rate (FDR) < 0.01. **(K)** Expression levels of *SHMT2* and *PSPH* mRNA in AML patients (data from BloodSpot database^3^). **(L)** The correlation analysis of *SHMT2* and *SRSF7* mRNA expression (left panel) and *PSPH* and *SRSF7* mRNA expression (right panel) in AML patients (TCGA dataset). **(M)** Kaplan–Meier survival analysis of SHMT2^4^ (left panel) and PSPH^5^ (right panel) in AML patients. SHMT2 expression levels categorized AML patients into distinct groups: low expression and high expression. PSPH expression levels categorized AML patients into distinct groups: low expression and high expression. Log-rank test. **(N)** Quantitative reverse transcription PCR was used to measure *SHMT2* and *PSPH* mRNA levels in MOLM-13 cells with Scr or SRSF7 shRNAs. **(O)** Schematic diagram of SHMT2 mRNA alternative splicing patterns. The SHMT2 mRNA comprises a total of eight exons. Upon the knockdown of SRSF7 expression, an intron retention event occurs between exon 2 and exon 3 of the SHMT2 mRNA, introducing a premature termination codon. **(P)** The retained intron was detected by reverse transcription PCR in MOLM-13 cells expressing Scr or SRSF7 shRNA2 (S7sh2#). SHMT2 WT, SHMT2 wild-type; SHMT2 RI, SHMT2 retained intron. ∗*p* < 0.05, ∗∗*p* < 0.01, and ∗∗∗*p* < 0.001.

SRSF7 expression was down-regulated to elucidate its function in AML. SRSF7 knockdown in MOLM-13, NB-4, KASUMI-1, and OCI-AML3 cells was achieved using two specific shRNAs, with down-regulation verified through quantitative reverse transcription PCR (Fig. S2A–D). Colony formation assays revealed a notable decrease in colony number in MOLM-13 (Fig. 1B; Fig. S2H), NB-4 (Fig. S2E, I), KASUMI-1 (Fig. S2F, J), and OCI-AML3 (Fig. S2G, K) cells following SRSF7 knockdown.

The suppression of SRSF7 led to a notable increase in cell apoptosis within MOLM-13 (Fig. 1C; Fig. S3D), NB-4 (Fig. S3A, E), KASUMI-1 (Fig. S3B, F), and OCI-AML3 (Fig. S3C, G) cells. Next, we investigated whether SRSF7 knockdown promoted AML cell differentiation. Flow cytometry analysis revealed increased CD11b expression on the cell surfaces of MOLM-13, NB-4, KASUMI-1, and OCI-AML3 (Fig. 1D; Fig. S4A–G), indicating that SRSF7 knockdown significantly promoted AML cell differentiation.

Cell cycle analysis also demonstrated that SRSF7 knockdown significantly reduced the S phase in MOLM-13 (Fig. 1E) and other AML cells (Fig. S5A–G) compared with the control scramble, indicating a marked decline in DNA replication ability.

SRSF7 knockdown hindered MOLM-13, NB-4, KASUMI-1, and OCI-AML3 cell proliferation compared with control scramble vector-transfected cells (Fig. 1F; Fig. S6A–C). Next, we further explored whether SRSF7 influenced the progression of AML *in vivo*. Figure S6D illustrates the expression of scramble and two SRSF7 shRNAs in MOLM-13 and KASUMI-1 cells. Following a three-day puromycin screening, the cells were transplanted into NKG immunodeficient mice via the tail vein. In the xenotransplantation models of MOLM-13 (Fig. 1G) and KASUMI-1 (Fig. S6E) cells, the survival time of the SRSF7 knockdown group was significantly longer.

RNA sequencing was used on AML cells with either the control scramble or SRSF7 shRNA to examine SRSF7's impact on AML. Sequencing results indicated that SRSF7 down-regulation influenced the expression of various genes. In the SRSF7 knockdown group, 673 genes were up-regulated and 442 were down-regulated (Fig. 1H). SRSF7 regulates the expression of various splicing-related genes, including other SRSF family members (SRSF1–6, SRSF8–10), Musashi RNA binding protein 2 (MSI2), and other associated factors. The Kyoto Encyclopedia of Genes and Genomes (KEGG) enrichment analysis revealed significant enrichment of cellular differentiation pathways among up-regulated differentially expressed genes (Fig. S7A). The down-regulated genes showed significant enrichment in the mitogen-activated protein kinase (MAPK) and Ras-associated protein-1 (Rap1) signaling pathways (Fig. S7B). The replicate multivariate analysis of transcript splicing (rMATS) analysis indicated that SRSF7 knockdown, compared with the control scramble, triggered numerous alternative splicing events: 5877 alternative 3′ splice sites (A3SS) and 1275 alternative 5′ splice sites (A5SS) (Fig. 1I, J).

KEGG analysis (Fig. S7B) indicated that steroid biosynthesis and glycine, serine, and threonine metabolism pathways exhibit the highest enrichment scores among down-regulated genes. The steroid biosynthesis pathway involves CYP2R, HSD17B7, LSS, MSMO1, and TM7SF2, while the glycine, serine, and threonine metabolism pathway includes CBS, CTH, PHGDH, PSAT1, PSPH, and SHMT2. Next, to find out the genes that play crucial roles in SRSF7-regulated pathways, we analyzed mRNA levels of these genes in AML patients and healthy donors (Fig. 1K; Fig. S8), and the association of these gene expression levels with SRSF7 (Fig. 1L; Fig. S9), as well as the association of their expression levels with the prognosis of AML patients (Fig. 1M; Fig. S10). The expression level of serine hydroxymethyltransferase 2 (SHMT2) and phosphoserine phosphatase (PSPH) in AML was significantly up-regulated compared with healthy controls (Fig. 1K). Additionally, their expression levels positively correlated with SRSF7 expression (Fig. 1L). A negative correlation between SHMT2/PSPH expression levels and patient outcomes was observed in the AML cohort (Fig. 1M). Quantitative reverse transcription PCR revealed that SRSF7 knockdown significantly reduced *SHMT2* mRNA expression, rather than *PSPH* mRNA (Fig. 1N). Alternative splicing analysis revealed that SRSF7 knockdown led to the retention of intron 2 in *SHMT2* mRNA, resulting in a premature termination codon and subsequent RNA degradation through the nonsense-mediated mRNA decay (NMD) pathway (Fig. 1O). Furthermore, the PCR results demonstrated that intron retention occurred in AML cells expressing SRSF7 shRNA (Fig. 1P). Collectively, the down-regulation of SRSF7 expression results in the retention of intron 2 within the *SHMT2* pre-mRNA as well as the dysregulation of other pathways, leading to cell death and cell cycle arrest, thereby inhibiting AML development (Fig. S11).

In summary, our research demonstrates that SRSF7 is overexpressed in AML patients, and its knockdown impedes AML cell proliferation *in vitro* and *in vivo*. SRSF7 contributes to AML development by regulating SHMT2 alternative splicing. This study has elucidated SRSF7's role in AML and identified new potential therapeutic targets for AML.

## CRediT authorship contribution statement

**Dantong Shang:** Investigation, Writing – original draft, Formal analysis, Data curation. **Hongkai Zhu:** Data curation. **Yulin Pu:** Investigation. **Long Liang:** Data curation. **Haodong Xu:** Data curation. **Yue Sheng:** Data curation, Funding acquisition, Writing – review & editing, Conceptualization, Methodology. **Huifang Zhang:** Funding acquisition, Methodology, Conceptualization, Writing – review & editing.

## Ethics declaration

The Animal Care and Use Committee of the Second Xiangya Hospital approved all animal experiments. The Second Xiangya Hospital of Central South University Institutional Review Board approved all samples involving humans (ethics approval number: Z0258-02). Informed consent was secured from all participants involved in the study.

## Funding

This work was partially supported by grants from the National Key R&D Program of China (No. 2024YFC2510500), the 10.13039/501100004735Hunan Natural Science Foundation of China (No. 2025JJ60563), the 10.13039/501100001809National Natural Science Foundation of China (No. 32270791), the Scientific Research Project of Hunan Provincial Health Commission (China) (No. C202303046332), the Scientific Research Project of Hunan Provincial Department of Education (China) (No. 23A0018), the Huxiang Youth Talent Support Program (China) (No. 2022RC1205), the 10.13039/501100001688International Centre for Genetic Engineering and Biotechnology (ICGEB) (No. CRP/CHN22-03_EC), and the American Society of Hematology.

## Conflict of interests

The authors declare that they have no known competing financial interests or personal relationships that could have appeared to influence the work reported in this paper.
