# Supplementary material for: SRSF7 serves as a potential therapeutic target in acute myeloid leukemia
Source: Genes Dis. 2025 Jun 26;13(2):101739. doi: 10.1016/j.gendis.2025.101739 (PMC12606998; doi:10.1016/j.gendis.2025.101739)
Supplement: Multimedia component 1 [file mmc1.docx]

**Methods and materials**

**Cell culture**

KASUMI-1 cell is cultivated in RPMI medium (20% fetal bovine serum, FBS), while MOLM-13, OCI-AML3, and NB-4 cells in RPMI medium (10% FBS). Each medium was fortified with 1% penicillin and streptomycin to ensure sterility. Peripheral blood from healthy donors was treated with red blood cell lysis buffer to collected leukocyte pellet for further analysis.

**Construction of shRNA Expression Vector**

Two human-specific short hairpin RNA (shRNA) primers targeting SRSF7 were meticulously designed utilizing the Broad Institute's online portal, ensuring precision in gene silencing studies. The sequence of shRNA1# and shRNA2# were listed in **Supplemental Table 1**. The vector for shRNA was PLKO.1.

**Lentivirus infection**

Lentiviral particles were generated by transfecting 293T cells with human SRSF7-targeting shRNAs, alongside packaging plasmids PSPAX.2 and PMD2.G, utilizing the polyethylenimine (PEI) method. The supernatant containing the viral particles was harvested at intervals of 24, 48, and 72 hours post-transfection to ensure optimal viral yield. The AML cell lines MOLM-13, NB-4, KASUMI-1, and OCI-AML3 were successfully transduced using viral supernatant, which was added with 5 μg/mL polybrene to enhance transduction efficiency. They were subjected to centrifugation at 2000 rpm for three hours daily over a period of three consecutive days to facilitate efficient transduction. Subsequent to transduction, the cells underwent a 72-hour selection with 2 μg/mL puromycin, thereby facilitating enrichment of cells that had been successfully transduced.

**RT-PCR and RT-qPCR Analysis**

Initially, cellular lysates were prepared using TRIzol reagent, followed by the extraction of total RNA through a phenol-chloroform protocol, ensuring the isolation of high-quality RNA for subsequent analyses. mRNA was then reverse transcribed using an RNA reverse transcription kit (AG11728). RT-qPCR was completed utilizing the SYBR Green (AG11701). Primers for the detection of *SRSF7*, *SHMT2*, and *GAPDH* mRNA levels were meticulously selected and are detailed in **Supplementary Table 1**, ensuring accurate and reproducible quantification of target transcripts. The analysis was conducted using a Light Cycler 96 qPCR instrument (Roche).

**Western blot**

Cells were lysed using the buffer with Protease Inhibitor Cocktail (Roche, catalog number 04693132001) to prevent degradation. Protein was separated using 10% SDS-PAGE. Next, protein was transferred to PVDF membrane, following by incubation with blocking liquid. Subsequently, it was incubated with primary antibodies SRSF7 (Proteintech, cat# 11044-1-AP). Membranes were incubated with secondary antibodies and quantified employing the ChemiDoc™ XRS system (Bio-Rad Laboratories Co., Ltd.).

**Colony formation assay**

MOLM-13, NB-4, KASUMI-1, and OCI-AML3, transfected with either a control scramble vector or two SRSF7 shRNAs, were harvested and plated at 4 x 10^3 cells/mL in triplicate into human methylcellulose base media (R&D, cat# HSC002) containing 2 μg/mL puromycin. The colony count was recorded 7 days post-plating.

**Flow cytometric analysis**

For cell apoptosis and differentiation analysis, AML cells (MOLM-13, NB-4, KASUMI-1, and OCI-AML3) transfected with control Scramble vector or two SRSF7 shRNAs were collected, washed twice in PBS, and stained with CD14 and CD11b cell surface markers, followed by DAPI and Annexin V staining. For the analysis of cell cycle, cells were cultured in 30 μg/ml BrdU. Subsequently, cells were treated sequentially with 2% formaldehyde, ethanol, and 0.1% Tween-20. DAPI and anti-BrdU antibodies were used to stain the cells. FACS CaliburTM flow cytometry was used to analyze all samples (BD Biosciences).

**Bone marrow transplantation**

NKG mice, NOD.Cg-PrkdcscidIl2rgem1cya/Cya, were purchased from Cyagen (China). 8-week-old irradiated NKG mice (1.5 Gray) were transplanted via tail vein injection with 5 x 10^4 MOLM-13 cells and 2 x 10^6 KASUMI-1 cells per mouse. The survival time of the mice were observed and recorded.

**Statistical analysis**

The results were mean ± standard deviation. Every study was conducted independently a minimum of three times to ensure reliability. Two groups were compared by two-tailed Student's *t*-test, while one-way ANOVA for multiple groups. Kaplan-Meier survival curve was created with GraphPad Prism.

**Supplementary figures**

**
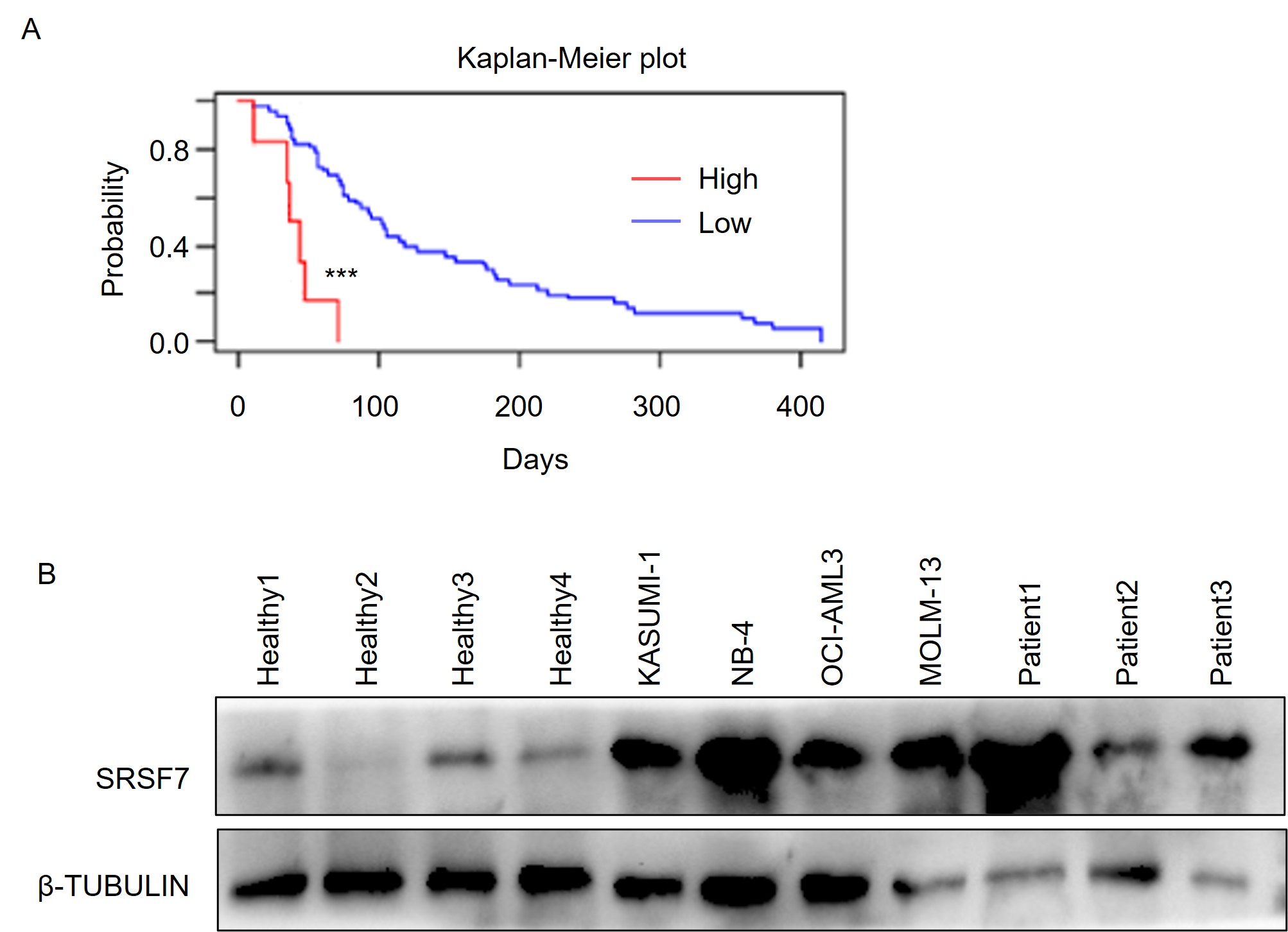
**

**Supplementary Figure 1: The aberrant overexpression of SRSF7 in patients with AML is notably correlated with an unfavorable prognosis. A**. Kaplan-Meier survival analysis of the GSE5112 dataset (*n* = 58). SRSF7 expression of AML patients stratified them into distinct cohorts: low expression and high expression, log-rank test. **B**. WB elucidated the SRSF7 protein level in AML cells as well as in peripheral blood mononuclear cells of AML patients and the healthy. One-way ANOVA: **P* < 0.05, ***P* < 0.01, ****P* < 0.001.


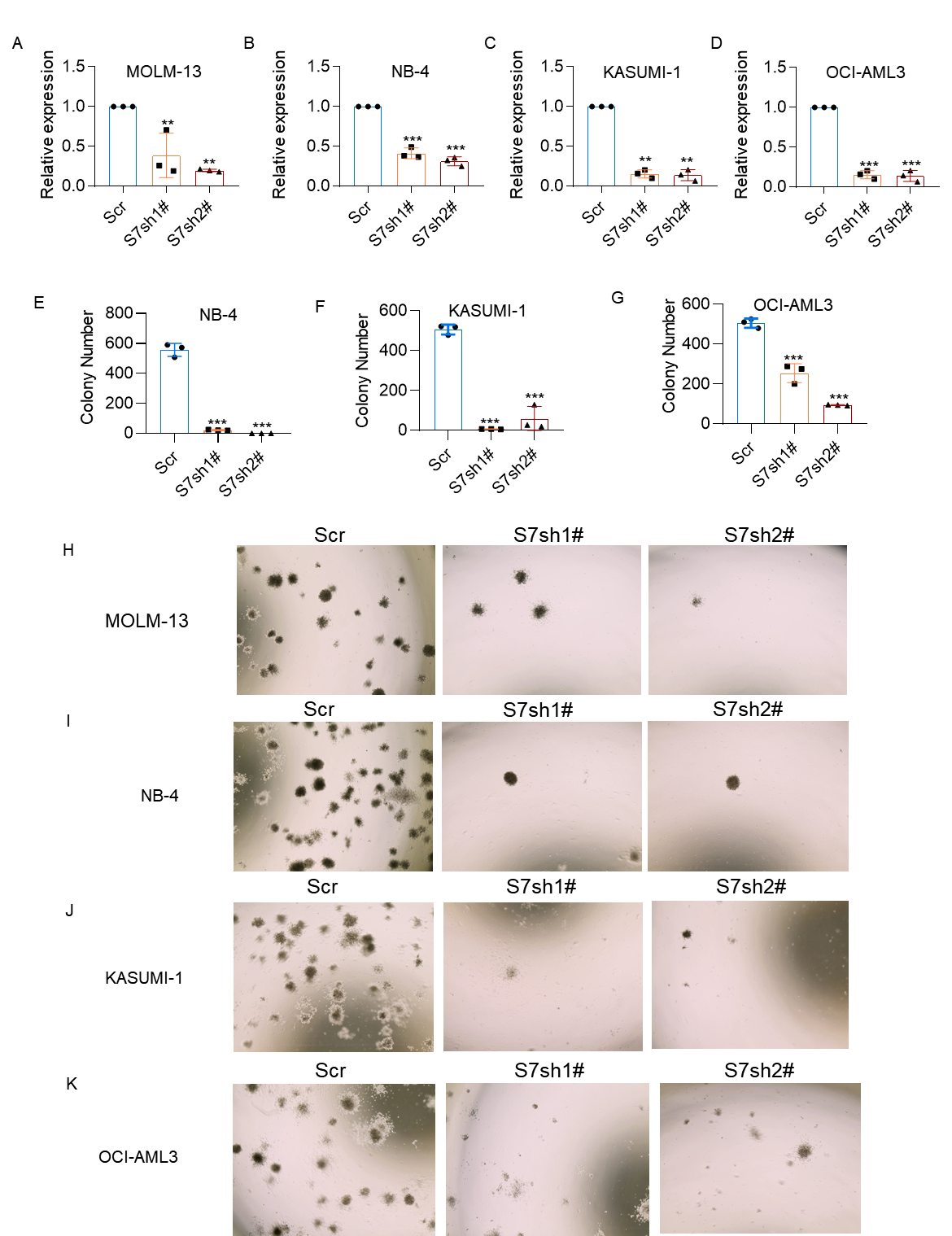


**Supplementary Figure2: SRSF7 knockdown impairs AML cell colony formation. A-D**. The knockdown effectiveness of Scramble (Scr), SRSF7 shRNA1 (S7sh1#), and SRSF7 shRNA2 (S7sh2#) in MOLM-13 (**A**), NB-4 (**B**), KASUMI-1 (**C**), and OCI-AML3 (**D**) cells was assessed by RT-qPCR. **E-K**. Colony formation of NB-4 (**E**), KASUMI-1 (**F**), and OCI-AML3 (**G**) cells expressing control Scramble or SRSF7 shRNAs was analyzed. Figures **H**, **I**, **J**, and **K** display representative images at 40 × magnification. 500 μL methylcellulose-based medium contained 2000 AML cells, and the resulting colony formation was quantitatively assessed after a 7-day incubation period. One-way ANOVA: ***P* < 0.01 and ****P* < 0.001.

**
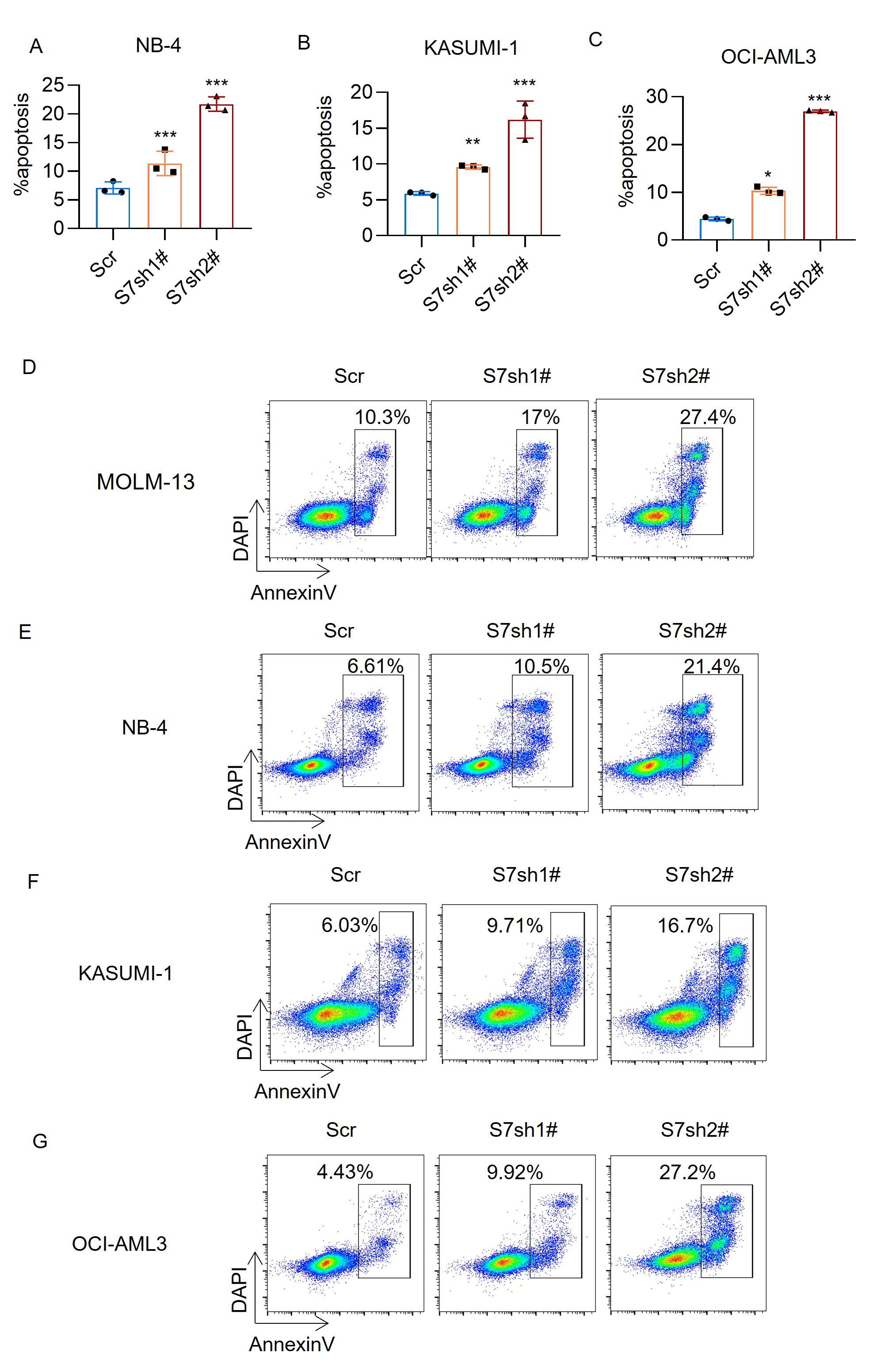
**

**Supplementary Figure 3: SRSF7 downregulation induces AML cell apoptosis.** Flow cytometry analyzed the apoptosis of NB-4 (**A**), KASUMI-1 (**B**), and OCI-AML3 (**C**) expressing control Scramble (Scr) or SRSF7 shRNAs. Figure **D**, **E**, **F**, and **G** represented the gating strategy. One-way ANOVA: **P* < 0.05, ***P* < 0.01, and ****P* < 0.001.


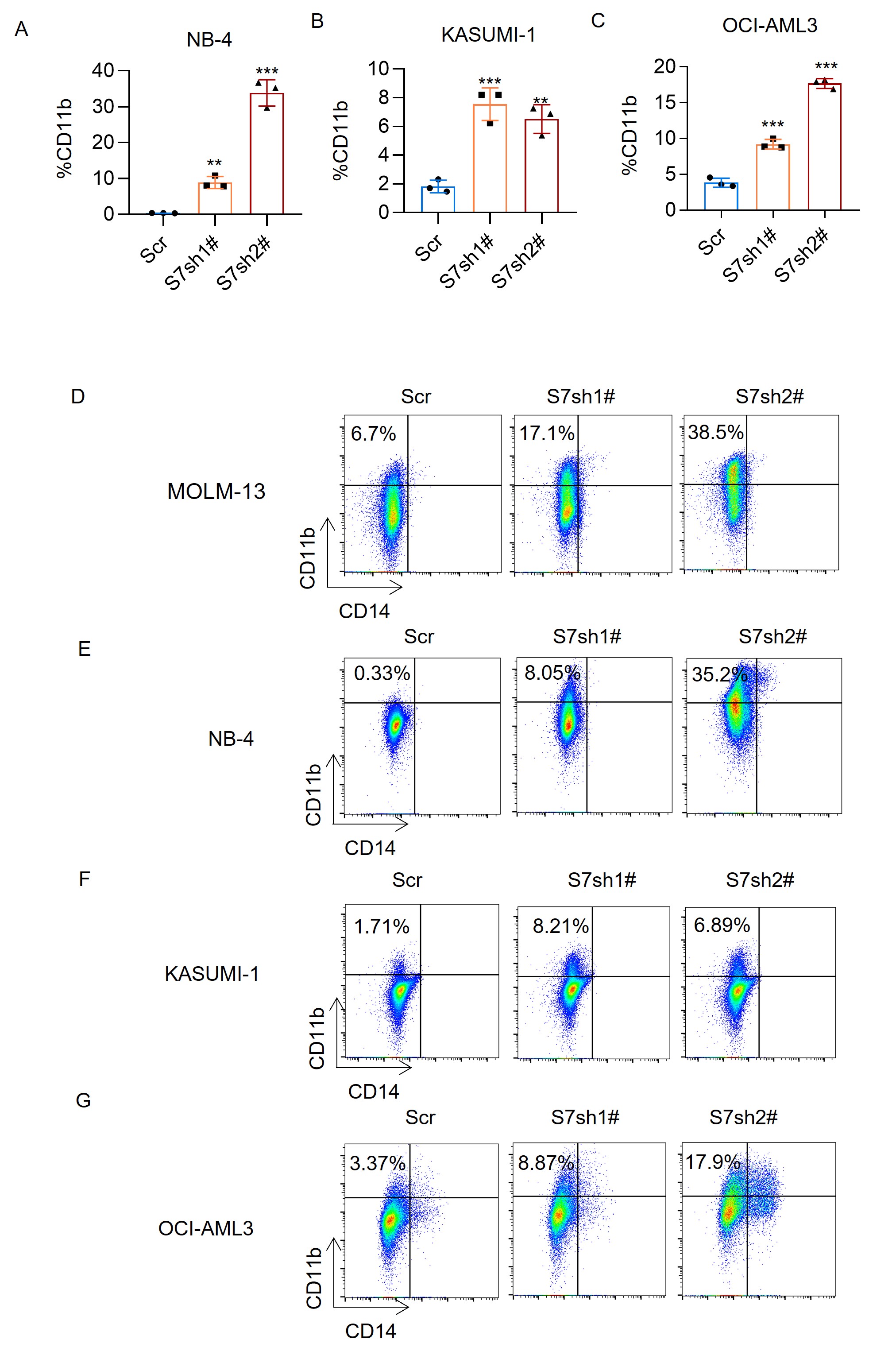


**Supplementary Figure 4: SRSF7 downregulation promotes AML cell differentiation.** Flow cytometry analyzed the ratio of CD11b positive cells NB-4 (**A**), KASUMI-1 (**B**), and OCI-AML3 (**C**) expressing control Scramble (Scr) or SRSF7 shRNAs. Figure **D**, **E**, **F**, and **G** were gating strategy. One-way ANOVA: ***P* < 0.01, ****P* < 0.001.


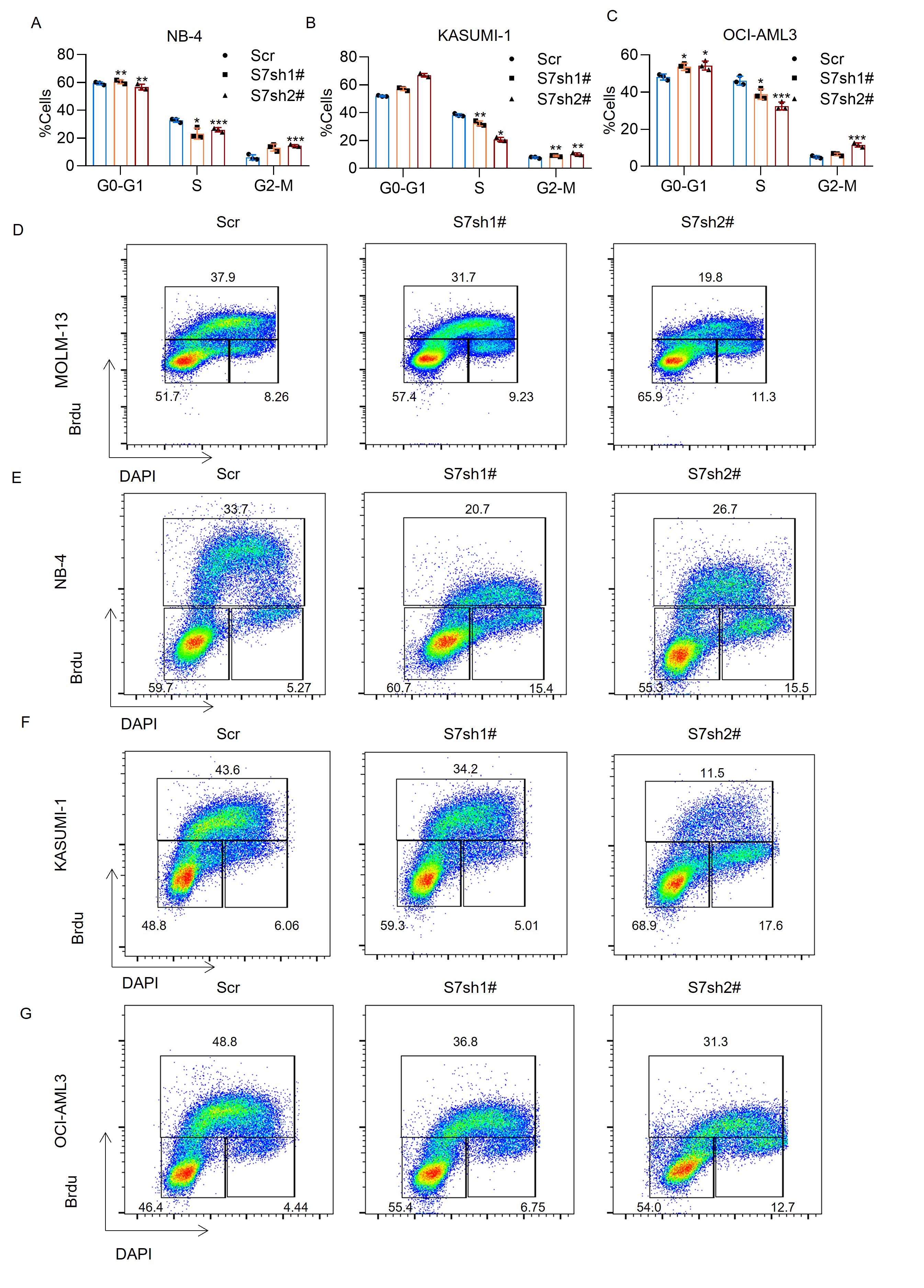


**Supplementary Figure 5: Downregulation of SRSF7 arrest cell cycle of AML cells.** Flow cytometry analyzed the cell cycle progression of NB-4 (**A**), KASUMI-1 (**B**), and OCI-AML3 (**C**) cells expressing control Scramble (Scr) or SRSF7 shRNAs. Figure **D**, **E**, **F**, and **G** represented the gating strategy. One-way ANOVA: ***P* < 0.01, ****P* < 0.001.


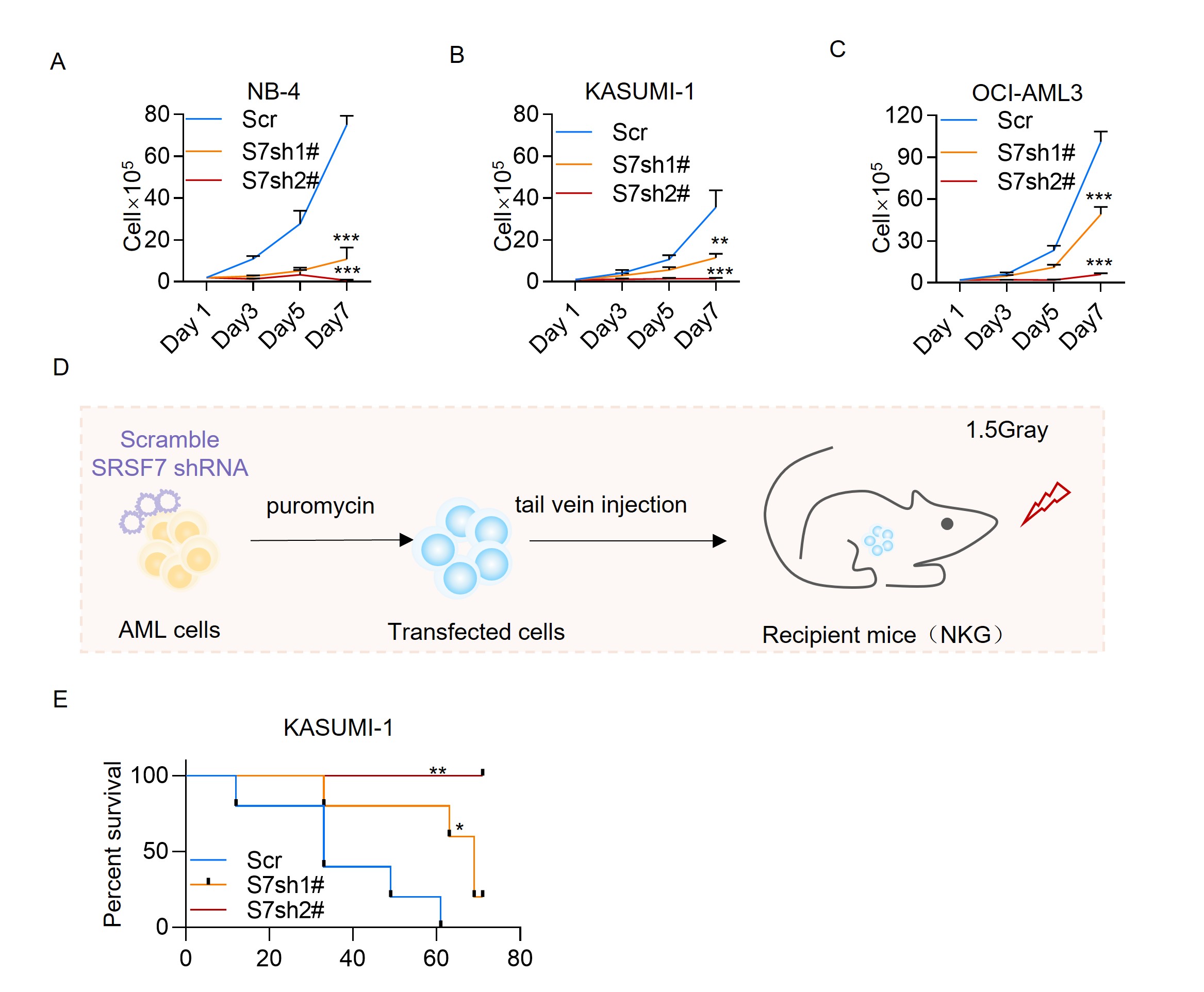


**Supplementary Figure 6: SRSF7 knockdown inhibited AML cell proliferation. A-C**. Growth curves of NB-4, KASUMI-1, and OCI-AML3 cells expressing control Scramble (Scr) or SRSF7 shRNAs, one-way ANOVA. **D**. Schematic depiction of the xenotransplantation assay. Post viral infection, AML cells with Scramble or SRSF7 shRNAs underwent a three-day puromycin treatment before being transplanted into 1.5 Gray-irradiated NKG mice (NOD.Cg-PrkdcscidIl2rgem1cya/Cya) via tail vein injection. **E.** Kaplan–Meier survival analysis was performed on immunodeficient mice transplanted with KASUMI-1 cells (n = 5 in each group) expressing either control Scramble (Scr) or SRSF7 shRNAs. Log-rank Test. **P* < 0.05, ***P* < 0.01, and ****P* < 0.001.


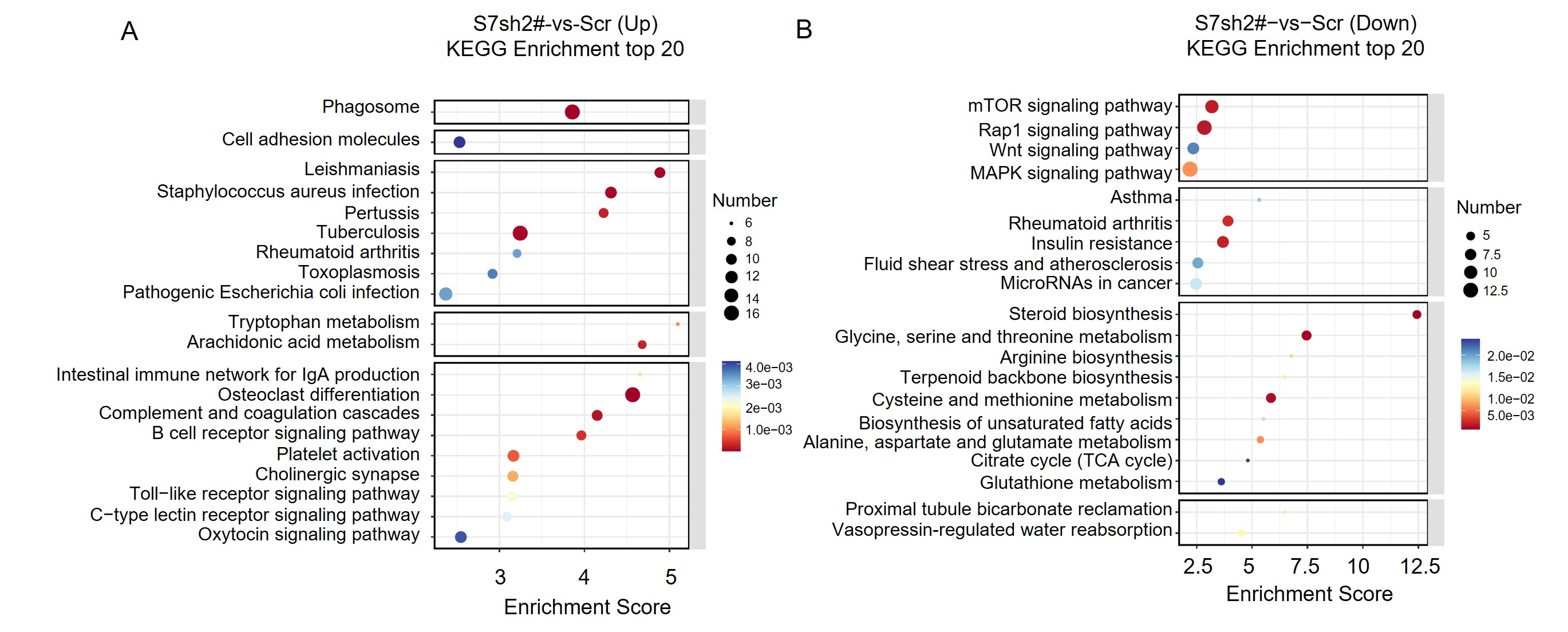


**Supplementary Figure 7: SRSF7 regulates multiple signaling pathways in AML.** **A-B**. Differentially expressed genes regulated in SRSF7 knockdown cells were analyzed by KEGG enrichment. Panel A displays top 20 upregulated pathways, while top 20 downregulated pathways were displayed in Panel B.


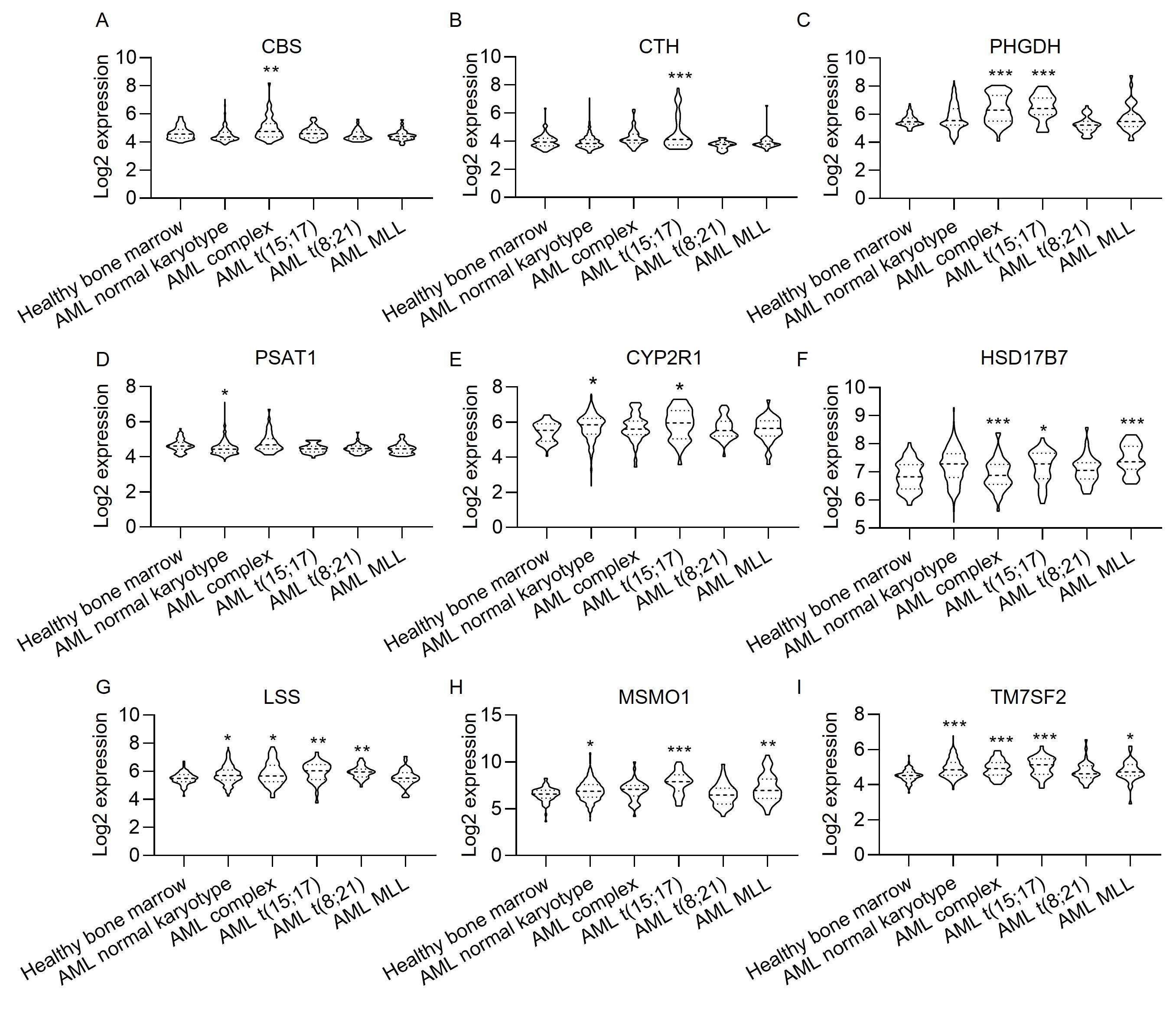


**Supplementary Figure 8.** The expression levels of genes involved steroid biosynthesis pathway and glycine, serine and threonine metabolism pathway (**A-I)** in the primary AML patients with different chromosome translocations were compared with those of healthy donors. One-way ANOVA: **P*< 0.05, ***P* < 0.01, ****P* < 0.001.


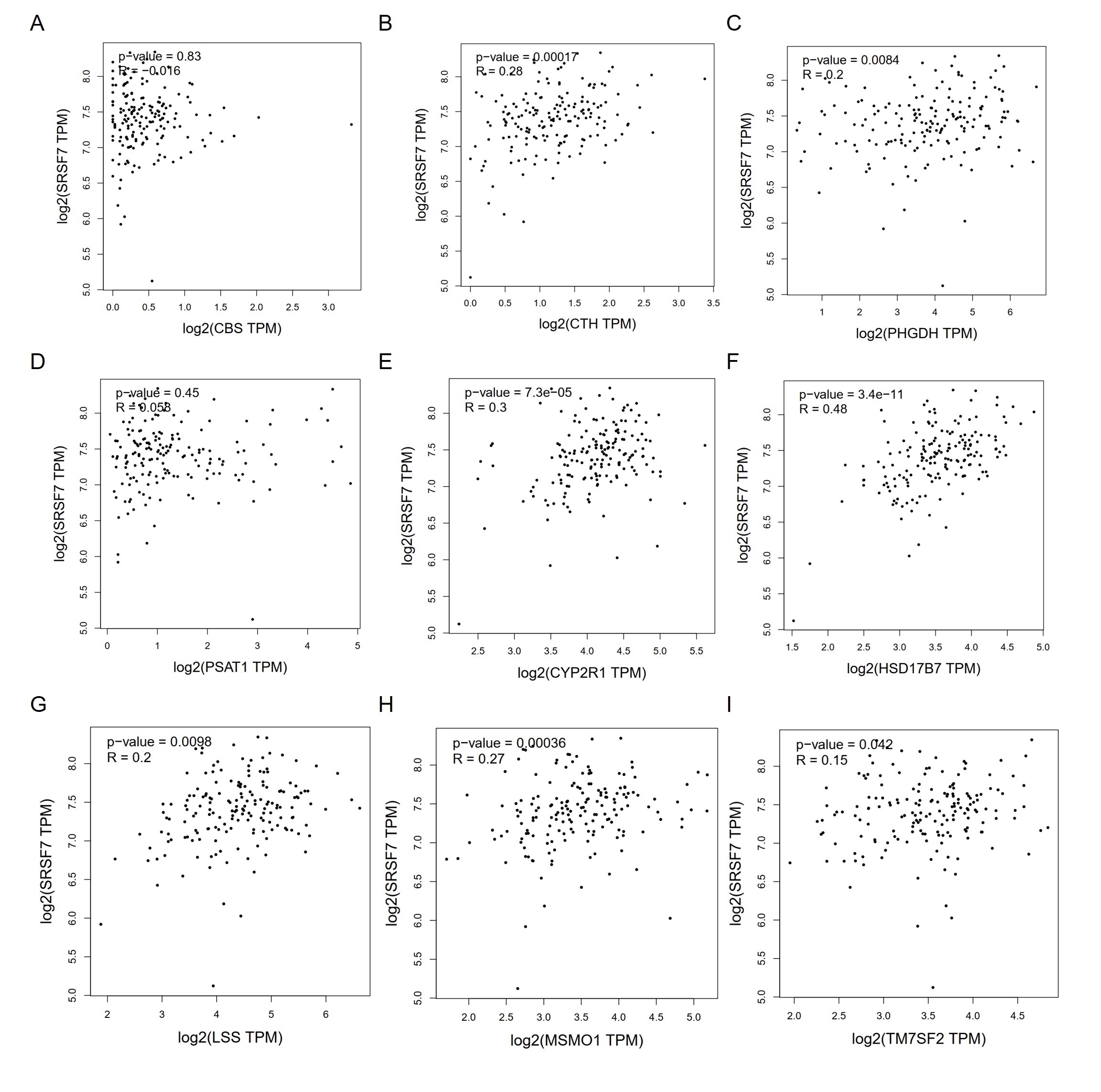


**Supplementary Figure 9.** Correlation of genes involved steroid biosynthesis pathway and glycine, serine and threonine metabolism pathway (**A-I**) and *SRSF7* mRNA expression in AML patients from TCGA dataset. The *P*-value was tested by Spearman Correlation Analysis.


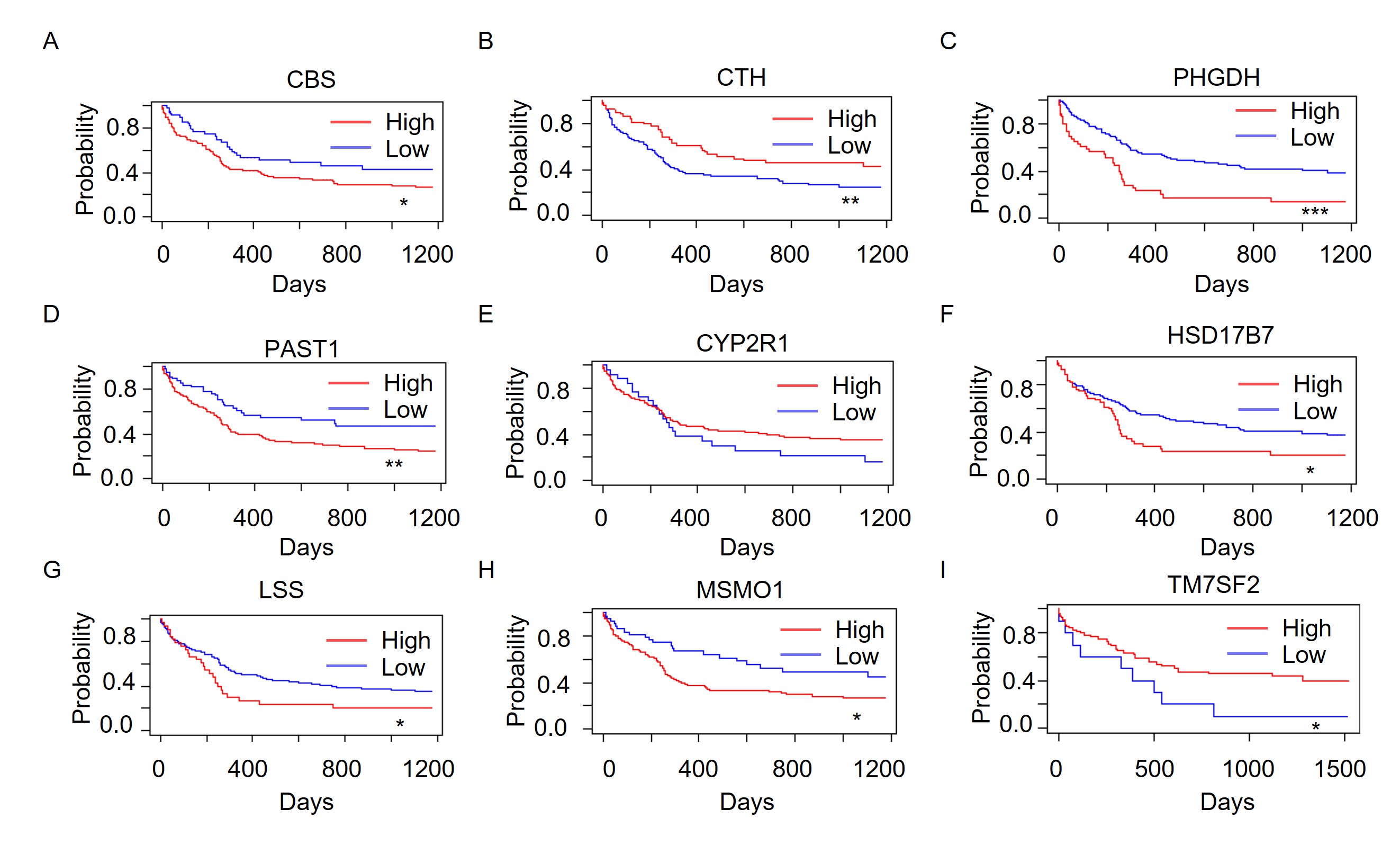


**Supplementary Figure 10.** Kaplan–Meier survival analysis of genes involved steroid biosynthesis pathway and glycine, serine and threonine metabolism pathway in AML patients. The expression levels of these genes categorized AML patients into distinct groups: low expression and high expression. Log-rank test: **P* < 0.05, ***P* < 0.01, ****P* < 0.001.


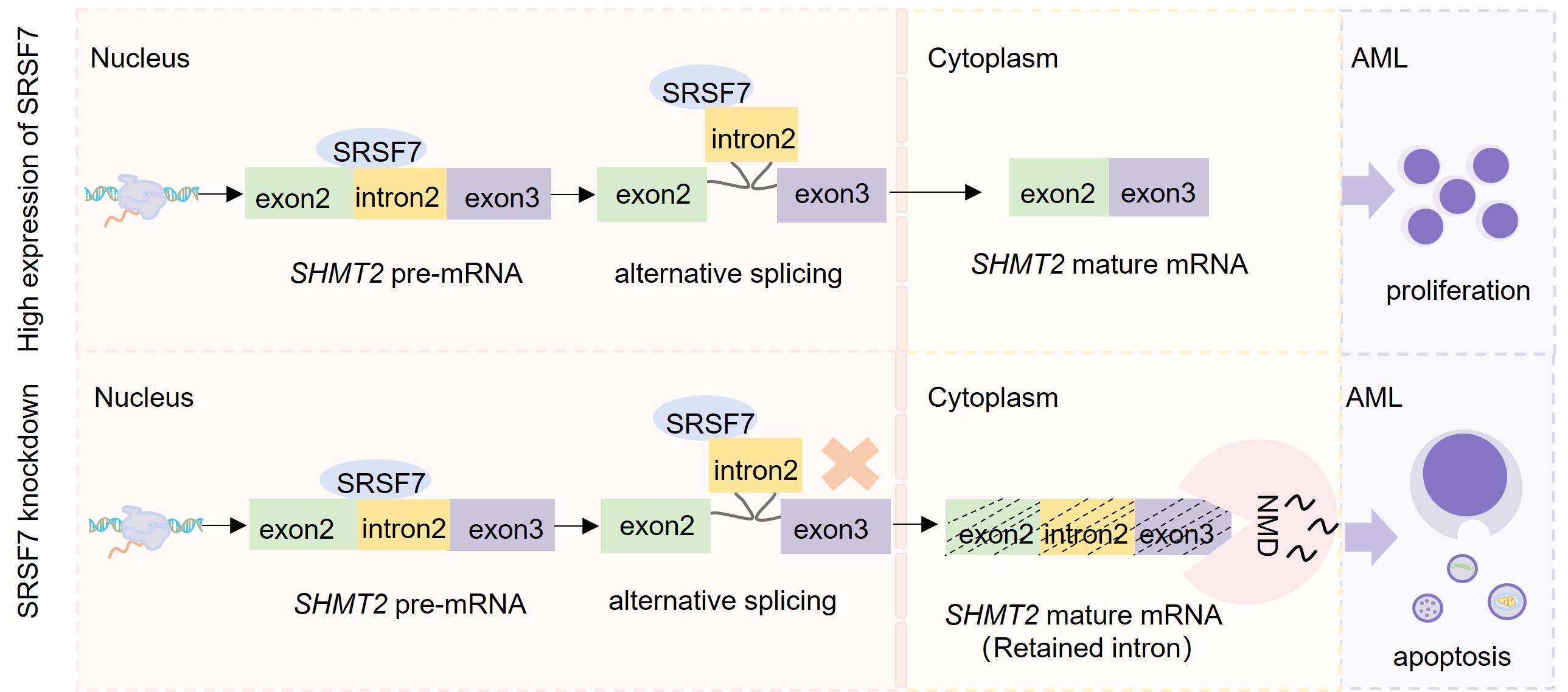


**Supplementary Figure 11: SRSF7 regulates alternative splicing of SHMT2.** In summary, elevated SRSF7 expression levels orchestrate the maturation of *SHMT2* mRNA within the nucleus by mediating the exclusion of intron 2 between exons 2 and 3 through alternative splicing. This promotes mature mRNA translation in the cytoplasm, enhancing AML cell proliferation. Conversely, the suppression of SRSF7 expression leads to intron retention between exons 2 and 3 in the *SHMT2* pre-mRNA, introducing a premature termination codon. NMD pathway subsequently degraded the mRNA in the cytoplasm, leading to AML cell apoptosis.

**Supplementary Table 1: Primer list**

| SRSF7 qpcr-F | CGCTGGCAAAGGAGAGTTAG |
| --- | --- |
| SRSF7 qpcr -R | CGAATTCCACAAAGGCAAAT |
| SHMT2 qpcr -F | CCCTTCTGCAACCTCACGAC |
| SHMT2 qpcr -R | TGAGCTTATAGGGCATAGACTCG |
| PSPH qpcr -F | GCATAAGGGAGCTGGTAAGTCG |
| PSPH qpcr -R | ACCTGCATATTCACCGTTAAAGT |
| GAPDH qpcr -F | GAGTCAACGGATTTGGTCGT |
| GAPDH qpcr -R | GACAAGCTTCCCGTTCTCAG |
| SRSF7 sh1#-F | CCGGGAACTGTATGGATTGCGAGAACTCGAGTTCTCGCAATCCATACAGTTCTTTTTG |
| SRSF7 sh1#-R | AATTCAAAAAGAACTGTATGGATTGCGAGAACTCGAGTTCTCGCAATCCATACAGTTC |
| SRSF7 sh2#-F | CCGGGATCAAGATCCAGGTCTATTTCTCGAGAAATAGACCTGGATCTTGATCTTTTTG |
| SRSF7 sh2#-R | AATTCAAAAAGATCAAGATCCAGGTCTATTTCTCGAGAAATAGACCTGGATCTTGATC |
| SHMT RI detection-F | TGGAGCTCATTGCCTCAGAG |
| SHMT RI detection-R | GCAGCTCGGCTGCAGAAGTT |
