# Supplementary material for: SRSF7 serves as a potential therapeutic target in acute myeloid leukemia
Source: Genes Dis. 2025 Jun 26;13(2):101739. doi: 10.1016/j.gendis.2025.101739 (PMC12606998; doi:10.1016/j.gendis.2025.101739)
Supplement: Multimedia component 2 [file mmc2.docx]

**Availability of data and Materials**

RNA-seq data has been deposited in NCBI with reference number (GSE272201).
