# Supplementary material for: SRSF7 serves as a potential therapeutic target in acute myeloid leukemia
Source: Genes Dis. 2025 Jun 26;13(2):101739. doi: 10.1016/j.gendis.2025.101739 (PMC12606998; doi:10.1016/j.gendis.2025.101739)
Supplement: Multimedia component 3 [file mmc3.pdf]

中南大学湘雅二医院实验动物福利伦理审查同意书

Approval of Animal Ethic

The Second Xiangya Hospital, Central South University

批准编号 Approval No.	20240236

本动物实验方案经过中南大学湘雅二医院实验动物伦理委员会审核，符合动物保护、动物福利和伦理原则，符合国家实验动物福利伦理的相关规定。The animal use protocol listed below has been reviewed and approved by the Institutional Animal Care and Use Committee (IACUC), The Second Xiangya Hospital, Central South University, China

**实验名称** Study Title	SRSF7通过调控SHMT2的可变剪接促进急性髓系白血病进展的作用与机制研究				
**实验申请人** Applicant	张慧芳	**职称/学位** Title/Degree	博士	**邮箱** Email	15580857265@163.com
**项目负责人** Principle Investigator (PI)	张慧芳	**职称/学位** Title/Degree	博士	**邮箱** Email	15580857265@163.com
**院系(部门)** Department	血液内科		**申请日期** Application date		2024-03-07 15:31
**动物种系** Species or Strains	小鼠		**动物数量** Quantity		21
**计划执行时间** Period of experiments	2025-01-01至2027-01-01		**实验动物使用许可证** License No. of Laboratory Animal Facility		SYXK（湘）2022-0012
**审查意见** Results from committee	**【 √ 】符合动物福利伦理要求，同意实验 Agree**<br>**【 】调整方案后，可进行实验 Agree after modification**<br>**【 】不同意 Disagree**				

中南大学湘雅二医院实验动物伦理委员会

Animal Ethical and Welfare Committee，The Second Xiangya Hospital, CSU，P.R.China

日期（Date）： 2024/03/07

中南大学湘雅二医院 实验动物伦理委员会
