# Supplementary material for: SRSF7 serves as a potential therapeutic target in acute myeloid leukemia
Source: Genes Dis. 2025 Jun 26;13(2):101739. doi: 10.1016/j.gendis.2025.101739 (PMC12606998; doi:10.1016/j.gendis.2025.101739)
Supplement: Multimedia component 4 [file mmc4.pdf]

中南大学湘雅二医院纵向项目申报伦理审批件

（2024）申报伦理审查（Z0258-02）号

<table>
<tr><td>项目名称</td><td colspan="3">SRSF7通过调控SHMT2的可变剪接促进急性髓系白血病进展的作用与机制研究</td></tr>
<tr><td>项目负责人</td><td>张慧芳</td><td>科室</td><td>血液内科</td></tr>
<tr><td>系列</td><td>医疗</td><td>职称</td><td>医师</td></tr>
<tr><td>申报项目类型</td><td colspan="3">■ 国自然 － 青年科学基金项目</td></tr>
<tr><td>研究内容</td><td colspan="3">■ 临床前研究 ■ 临床研究</td></tr>
<tr><td rowspan="2">临床前研究</td><td>■ 生物样本</td><td colspan="2">涉及人体细胞或组织采集： ■ 是 □ 否
采集（ 15 ）例人细胞或是组织
种类： ■ 血细胞
部位： ■ 其他 （血液）部位

涉及既往生物样本： □ 是 ■ 否</td></tr>
<tr><td>■ 动物</td><td colspan="2">■ 是（由动物伦理委员会负责审批） □ 否</td></tr>
<tr><td rowspan="2">临床研究</td><td colspan="3">■ 非干预性： ■ 病历资料回顾性研究 ■ 其他类型 仅用细胞进行科研实验</td></tr>
<tr><td colspan="3">□ 干预性： 无</td></tr>
<tr><td>承担责任</td><td colspan="3">中南大学湘雅二医院作为： ■ 组长单位 □ 参与单位</td></tr>
<tr><td>合作单位</td><td colspan="3">□ 有 ■ 无</td></tr>
<tr><td>项目主管部门审核意见</td><td colspan="3">同意该项目进行申报。
经办： 科研部
日期：2024年03月10日</td></tr>
<tr><td>伦理委员会意见</td><td colspan="3">经伦理初步审核，同意该项目进行申报。项目在相关部门立项后到签订合同书前请经项目主管部门初审后再次递交伦理会议审查申请，逾期将不予受理。所有涉及人的临床研究均需经过伦理审核批准方可开展。

2024年03月10日
中南大学湘雅二医院临床研究伦理委员会盖章</td></tr>
</table>
